# Hearing Loss in Workers Exposed to Toluene and Noise

**DOI:** 10.1289/ehp.8959

**Published:** 2006-04-26

**Authors:** Shu-Ju Chang, Chiou-Jong Chen, Chih-Hui Lien, Fung-Chang Sung

**Affiliations:** 1 Department of Industrial Management, Aletheia University, Taipei, Taiwan; 2 Department of Environmental Engineering, National Ilan University, Ilan, Taiwan; 3 Institute of Occupational Safety and Health, Council of Labor Affairs, Executive Yuan, Taipei, Taiwan; 4 Institutes of Environmental Health, National Taiwan University College of Public Health, Taipei, Taiwan; 5 Institute of Environmental Health, China Medical University College of Public Health, Taichung, Taiwan; 6 Institutes of Preventive Medicine, National Taiwan University College of Public Health, Taipei, Taiwan

**Keywords:** adhesive products manufacturing, pure-tone audiometry, toluene, work-related hearing loss

## Abstract

In this study we investigated the risk of hearing loss among workers exposed to both toluene and noise. We recruited 58 workers at an adhesive materials manufacturing plant who were exposured to both toluene and noise [78.6–87.1 A-weighted decibels; dB(A)], 58 workers exposed to noise only [83.5–90.1 dB(A)], and 58 administrative clerks [67.9–72.6 dB(A)] at the same company. We interviewed participants to obtain sociodemographic and employment information and performed physical examinations, including pure-tone audiometry tests between 0.5 and 6 kHz. A contracted laboratory certified by the Council of Labor in Taiwan conducted on-site toluene and noise exposure measurements. The prevalence of hearing loss of ≥25 dB in the toluene plus noise group (86.2%) was much greater than that in the noise-only group (44.8%) and the administrative clerks (5.0%) (*p* < 0.001). The prevalence rates were 67.2, 32.8, and 8.3% (*p* < 0.001), respectively, when 0.5 kHz was excluded from the estimation. Multivariate logistic regression analysis showed that the toluene plus noise group had an estimated risk for hearing loss ≥25 dB, 10.9 times higher than that of the noise–only group. The risk ratio dropped to 5.8 when 0.5 kHz was excluded from the risk estimation. Hearing impairment was greater for the pure-tone frequency of 1 kHz than for that of 2 kHz. However, the mean hearing threshold was the poorest for 6 kHz, and the least effect was observed for 2 kHz. Our results suggest that toluene exacerbates hearing loss in a noisy environment, with the main impact on the lower frequencies.

Premature hearing loss in industrial workers is a well-known outcome of noise exposure at work. Pryor et al. (1983) and Rebert et al. (1983) were the first to suggest that organic solvents also present an ototoxic effect in animal studies. Barregard and Axelsson (1984) further suggested the possibility of an interaction between solvents and noise intensifying hearing loss in workers. Studies were subsequently conducted to examine the loss of auditory sensitivity due to organic solvents such as toluene, xylene, styrene, *n*-hexane, trichloro-ethylene, carbon disulfide, petroleum, and mixed solvents (Chang et al. 2003; Morata et al. 1993, 1995, 1997, 2002; Morioka et al. 2000; Sliwinska-Kowalska et al. 2001, 2004; Starck et al. 1999).

Attention has also been focused on toluene because it is an organic solvent widely used in various manufacturing industries. The impairment from toluene exposure or simultaneous exposure to both toluene and noise has been established in animal models (Johnson et al. 1990; Lataye and Campo 1997; Lataye et al. 1999; McWilliams et al. 2000). Regarding human exposure to toluene, Morata et al. (1993) found that workers at a printing plant with exposure to both toluene and noise experienced 11 times greater risk for hearing loss at ≥ 25 dB.

Morata et al. (1995) reviewed the deleterious effect of toluene exposure on hearing function and identified gaps for further study. Schaper et al. (2003) conducted a follow-up study over 5 years with 333 male workers in rotogravure printing plants. With the mean (± SD) lifetime weighted average exposures to 45 ± 17 ppm toluene and 82 ± 7 A-weighted decibel [dB(A)] noise in the past and 26 ± 20 ppm toluene and 82 ± 4 dB(A) noise during the study, they found no significant effect of toluene in hearing loss. Therefore, information is especially scarce on the threshold level of significant effect.

In the present study, more than half of the participating workers were employed at a plant that manufactured adhesive material and were exposed to toluene and/or noise for at least 10 years. The objective of this study was to evaluate the long-term effects of combined exposure to toluene and noise on audiometric thresholds.

## Materials and Methods

### Study subjects and data collection

We used a cross-sectional design that included interviewing participants and measuring environmental exposure and hearing function in a plant manufacturing adhesive materials using toluene as a solvent. All workers in the adhesive materials manufacturing section with exposure to both toluene and noise were men, and all were invited to participate in this study; 58 men participated (response rate, 89.2%). We used two reference groups: 58 male workers who worked in other sections of the plant and were exposed to noise only (response rate, 86.6%), and 58 male administrative clerks from the same company (response rate, 93.5%). Each participant provided informed consent, completed a questionnaire for information on health history and lifestyle, and underwent a health examination and hearing test required by Taiwan labor law. This study was approved by the company and by the Council of Labor Affairs Institute of Occupational Safety and Health. No personal data were published.

### Toluene exposure assessments

On-site environmental toluene samples were collected and measured using U.S. National Institute for Occupational Safety and Health (NIOSH) method 1500 (Eller 1994; NIOSH 1984) by a contracted laboratory certified by the Taiwan Council of Labor. Air samples were collected for three divisions: adhesive materials manufacturing division, application division, and recovery division. The on-site environmental air was pumped through a tube filled with 100 mg/50 mg (primary/backup) activated charcoal with a flow rate of 20–200 mL/min. The adsorption tube samples were sealed using plastic caps, stored on ice, and sent for analysis. Samples were desorbed using carbon disulfide and analyzed using an HP 5890 gas chromatograph/flame ionization detector (Hewlett-Packard, Avondale, PA, USA) to measure the toluene levels in samples. Seven samples were collected from the breathing zone for each division.

### Hearing test and noise assessment

A soundproof booth built by the Institute of Occupational Safety and Health (IOSH), the Council of Labor, Taiwan, was assembled in a quiet area in the administrative office. A physician conducted the otopharyngeal examination to screen for otitis and other otopathy for exclusion from the study. Audiograms were collected by an audiologist who was blinded to the participants’ subject group. All participants received pure-tone audiometry tests with a Beltone 2000 audiometer (Beltone Co., Chicago, IL, USA). Both ears were tested using the method of ascending at 1, 2, 3, 4, and 6 kHz and then descending to 1 and 0.5 kHz, following IOSH (1999) requirements; the test for 1 kHz was repeated. Frequency spectrum calibration in decibel hearing level fulfilled the International Organization for Standardization (ISO) 8253-1 criteria for audiometric testing environment (ISO 1989) adapted to the American National Standards Institute (ANSI) S 3.6-1968 requirement (ANSI 1970). Each person received the test 14 hr after the end of the previous work day. Daily calibration checks were conducted before subjects were tested.

On-site environmental noise levels for areas of the three study groups were assessed using sound pressure level meters (model B&K 2260; Bruel and Kjaer, Naerum, Denmark) based on the Taiwan Council of Labor requirements (IOSH 1999). Noise levels were measured in various locations throughout the study areas as well as in the same locations where air samples were collected for the toluene-exposed divisions. The measurements showed noise levels ranging from < 70 dB(A) to 90 dB(A) among the study areas. Most of the noise in the toluene plus noise group and the noise-only group was continuous. Time-weighted averages of noise levels were calculated for each group. Electroacoustic calibration was performed before data collection.

### Statistical analyses

We compared selected sociodemographic and lifestyle variables among the three study groups to identify potential confounding factors. The prevalence of hearing loss was calculated in percentage distribution based on the worse ear. The prevalence of hearing loss ≥25 dB and age-adjusted odds ratios (ORs) and 95% confidence intervals (CIs) of hearing loss ≥25 dB were estimated for the toluene plus noise and noise-only groups, using the administrative workers as the reference. Workers in the toluene plus noise group were also stratified by toluene exposure levels (average, 33.0 ppm in toluene recovery division, 107.6 ppm in the adhesive materials manufacturing division, 164.6 ppm in the adhesive application division). Average hearing loss levels were also calculated for each group. Because of a less reliable threshold for 0.5 kHz, we calculated the prevalence of and the OR for hearing loss ≥25 dB among the study groups using two models: the pure tone of 0.5 kHz was included in model 1 but excluded from model 2.

To differentiate the pure-tone impact among study subjects, we also plotted the mean hearing thresholds at frequencies of 1, 2, 3, 4, and 6 kHz and compared these plots among the study groups (Morata et al. 2002; Sliwinska-Kowalska et al. 2004). The toluene plus noise group was further stratified into subgroups based on the environmental noise levels [< 85 dB(A) and ≥ 85 dB(A)].

In order to estimate the dose–response effect of toluene on hearing loss for workers exposed to toluene plus noise, we calculated the cumulative exposure index (CEI) of toluene for each person in this group. The CEI was the product of the average toluene level in each division multiplied by the years of employment given as year-ppm. For example, an individual who had worked in the division for 10 years and had an average toluene level of 164.6 ppm received a 1,646 year-ppm cumulative exposure. We estimated and plotted the prevalence rates of hearing loss at 25–39, 40–54, and ≥55 dB and the mean hearing loss by stratified CEI.

To estimate the potential exposure threshold leading to a significant hearing loss, multivariate logistic analysis was performed to evaluate the dose–response effect based on CEI quartile distribution. Results of comparisons are reported with the statistical significance set at the 0.05 level. Data analyses were performed with SAS software (version 8.2; SAS Institute Inc., Cary, NC, USA).

## Results

There was no significant difference in age among the three study groups, with an average age range of 40.0–41.5 years (Table 1). Administrative clerks had received more school education (*p* < 0.001) but had a shorter employment history (*p* = 0.07). Approximately 28% of the workers exposed to toluene plus noise had worked for ≥20 years. The average noise exposure levels were 83.9 dB(A) in the toluene plus noise sites, 85.0 dB(A) in the noise-only sites, and 70.0 dB(A) in the administrative offices. Fewer than 15% of workers with noise exposure used hearing protectors.

The prevalence of hearing loss ≥25 dB was much greater in the toluene plus noise group (86.2%) than in the noise-only (44.8%) and administrative (5.0%) groups in model 1, when 0.5 kHz was included in the measurement (*p* < 0.001) (Table 2). The prevalence rate dropped approximately 20% for the toluene plus noise group when 0.5 kHz was excluded (model 2), but the difference in prevalence of hearing loss at ≥25 dB between these two groups remained large (67.2% vs. 32.8%, *p* < 0.001). Compared with the administrative clerks, the age-adjusted OR for hearing loss at ≥25 dB for all toluene plus noise–exposed workers was 7.7 and 4.2 times greater than that for the noise-only group in models 1 and 2, respectively. The OR of hearing loss at ≥25 dB among workers exposed to toluene plus noise increased as the toluene and noise level increased in model 1; however, the relationship was reversed in model 2.

**Figure 1:**
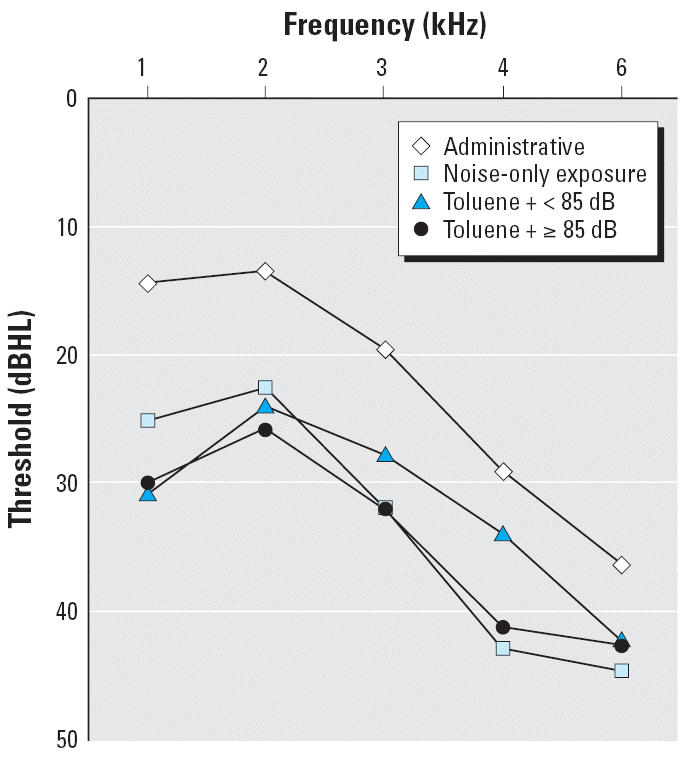
Group mean hearing thresholds [decibel hearing level (dBHL)] at frequencies between 1 and 6 kHz for administrative, noise-only, and toluene plus noise groups.

When we calculated the mean hearing thresholds at the measured pure-tone frequencies for each group, results showed a reversed J-shape with a turning point at the frequency of 2 kHz (Figure 1). Poorer hearing thresholds were observed at both low and high frequencies in the exposure groups, with the poorest at 4 and 6 kHz in both the toluene plus noise group and the noise-only group. The mean thresholds at higher frequencies were similar between the subgroup of toluene plus ≥85 dB and the noise-only group. The effects at 3 and 4 kHz were less for the subgroup of toluene plus < 85 dB. However, as for the 1 kHz frequency, workers in the toluene plus noise group had poorer thresholds than did those exposed to noise only.

**Figure 2:**
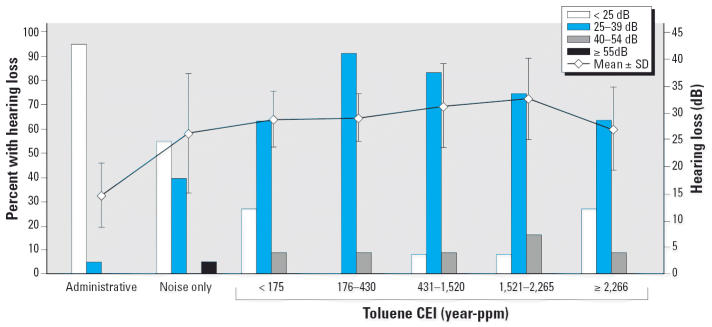
Comparison of hearing loss among the administrative, noise-only, and toluene plus noise groups by exposure and toluene CEI using model 1.

Figure 2 shows the prevalence rates of hearing loss with the inclusion of 0.5 kHz (model 1) and with the toluene plus noise group being stratified into quintile groups by the CEI of toluene. Hearing loss at 25–39 dB was most prevalent for the toluene plus noise group, with a peak prevalence at the CEI of 176–430 year-ppm toluene. The average hearing loss increased to a peak of 32.6 dB for those with exposures of 1,521–2,265 year-ppm toluene.

After controlling for age, smoking tobacco, drinking alcohol, and hearing protector use, the multivariate logistic regression analysis demonstrated an overall OR of 140 (95% CI, 32.1–608) for hearing loss in workers exposed to both toluene and noise in model 1 (0.5 kHz included) (Table 3). This analysis stratified the toluene plus noise group into four levels using the CEI of toluene. The hearing loss prevalence was 100% for workers with the CEI exposure of 201–530 year-ppm: the ORs showed a V-shape with an extreme risk at this toluene exposure level. The overall estimated risk for hearing loss dropped greatly to an OR of 29.1 (95% CI, 9.3–91.4) when 0.5 kHz was not used in the risk measure (model 2). The risk of hearing loss remained at a peak value at the exposure of 200–530 year-ppm but was much smaller (OR = 55.6; 95% CI, 9.7–317).

## Discussion

Previous human studies on the ototoxic effect of toluene from occupational exposure are not conclusive. Since the ototraumatic interaction between solvent and noise exposure was suggested by Barregard and Axelsson (1984), the effects have been assumed to be dependent on the exposure dose and period. A series of animal studies have demonstrated clear evidence of ototoxic effects with a very high level of toluene exposure over a short period of time (Johnson et al. 1990; Lataye and Campo 1997; Lataye et al. 1999; McWilliams et al. 2000). In humans, limited studies on this type of ototoxic effect have been conducted in occupational settings (Abbate et al. 1993; Morata et al. 1993, 1995, 1997; Schaper et al. 2003). However, the dose–response relationship for hearing loss had not been established in these studies.

With simultaneous exposure to toluene and noise, the prevalence rate of hearing loss in workers at a printing and paint manufacturing plant was lower in the study of Morata et al. (1993) than in the present study, even based on high frequency sounds (53% vs. 84%). The risk for hearing loss at ≥25dB was also much greater in the present study than in their study. Our study participants were older (40.0 vs. 32.5 years on average) and had a longer work history (12.3 vs. 8.1 years on average). In another study by Morata et al. (1997), their participants were also younger and had shorter work histories than in our study. They may have had less cumulative exposure to toluene. Many of our study participants had a longer employment history. This may explain why the rate of hearing loss was also profound in the noise-exposed group.

To our knowledge, the present study is the first to identify such a strong effect of hearing impairment from simultaneous exposure to toluene and noise in humans. In this study, the average noise exposure levels were similar between the toluene plus noise group and the noise-only group. However, the risk for hearing loss at ≥25 dB was much greater in the toluene plus noise group than in the noise-only group. The overall ORs adjusted for covariates were 140 versus 12.8 with 0.5 kHz included in the measurement (model 1) and 29.1 versus 5.0 with this pure tone excluded (model 2). This indicates that the risk for hearing loss boosted by toluene exposure may be more than six times greater than the risk induced by noise only.

The other unique finding in this study is that the magnitudes of ototoxic effect were different for various tested pure-tone frequencies among workers exposed to toluene plus noise, noise only, and administrative clerks. This finding has not been reported previously for toluene. It is worthwhile to note that the patterns of hearing impairment, measured by the pure-tone frequencies, associated with toluene plus noise exposure are similar to those associated with the simultaneous exposure to carbon disulfide and noise (Chang et al. 2003). Both toluene and carbon disulfide have greater impact on the speech frequencies than does noise alone, with the gap the largest at the frequency of 500 Hz. Therefore, the toluene plus noise group had poorer thresholds than did the noise-only group at 1 kHz frequencies, but not necessarily at high frequencies. However, the poorest mean hearing threshold in the toluene plus noise group was at 6 kHz. This was similar to the mean hearing threshold pattern found for the ototoxicity of styrene (Morata et al. 2002). We suspect that other types of ototoxic solvents may have other types of effects on hearing measured by pure-tone frequency.

The average air concentrations of toluene at work sites for the three divisions of the toluene plus noise group were 33.0 ppm, 107.6 ppm, and 164.6 ppm, but with similar noise exposure levels. It was surprising to find that the risk for hearing loss in workers with the lowest toluene exposure was only slightly lower than that for those with higher levels of toluene exposure. The dose–response analysis based on measures of toluene CEI showed a peak effect at the cumulative exposure level of 200–530 year-ppm and failed to estimate the threshold dose of toluene on the hearing loss effect due to the solvent. This observation might reflect variations in exposure history and healthy worker effect. Most of our study participants in the toluene plus noise group (all three areas) may have been exposed to higher levels of toluene during their long employment. Those who had a CEI > 200–530 year-ppm may have quit their jobs because of hearing problems or other reasons, which would lower the estimated ORs. This is one of the limitations of this study. Another limitation of this study was the sample size. No data were available for estimating the impact of hearing loss for workers due to toluene-only exposure.

Results from this study showed that there was an elevated hearing impairment for workers who were exposed to toluene plus noise compared with those exposed to noise alone. Although the overall hearing loss was rarely > 55 dB, the impact was greater for the speech frequencies than for the higher frequencies. These data suggest that the current work site threshold limit value of 100 ppm established for toluene does not protect workers from hearing loss in the simultaneous presence of noise at the work site. Effective intervention is needed to improve industrial safety of individuals experiencing ototoxic effects of solvents. Findings from this study and studies of other solvents can help policy makers as they establish threshold limit values for solvents and implement such interventions.

## Figures and Tables

**Table 1:** Selected characteristics of the study population [no. (%)] by study group.

	Exposure group	Reference group		
Variable	Toluene + noise (*n* = 58)	Noise only (*n* = 58)	Administrative (*n* = 60)	*p*-Value
Age (years)^a^	40.0 ± 9.7	41.5 ± 3.1	40.9 ± 3.4	0.418
< 40	22 (37.9)	16 (27.6)	24 (40.0)	
40–49	29 (50.0)	38 (65.5)	32 (53.3)	
≥50	7 (12.1)	4 (6.9)	4 (6.7)	
Education (years)				< 0.001
≤9	26 (44.8)	24 (41.4)	16 (26.6)	
10–12	30 (51.7)	30 (51.7)	14 (23.3)	
≥13	2 (3.4)	4 (6.9)	30 (50.1)	
Marital status				0.023
Unmarried	16 (25.8)	4 (6.9)	7 (11.7)	
Married	43 (74.1)	54 (93.1)	53 (88.3)	
Employment (years)^a^	12.3 ± 8.81	11.5 ± 5.73	9.52 ± 5.26	0.071
1–9	24 (41.4)	22 (37.9)	27 (45.0)	
10–19	18 (31.0)	31 (53.4)	30 (50.0)	
≥20	16 (27.6)	5 (8.6)	3 (5.0)	
Smoking tobacco				0.794
No	18 (31.0)	19 (32.8)	20 (33.3)	
Yes	40 (69.0)	34 (58.6)	36 (60.0)	
Quit	0 (0.0)	5 (8.6)	4 (6.7)	
Drinking alcohol				0.335
No	34 (58.6)	25 (43.1)	27 (45.0)	
Yes	17 (29.3)	24 (41.4)	28 (46.7)	
Quit	7 (12.1)	9 (15.5)	5 (8.3)	
Noise level				< 0.001
dB(A)^a^	83.9 ± 1.3	85.0 ± 4.2	70.0 ± 1.1	
Range	78.6–87.1	83.5–90.1	67.9–72.6	
Use hearing protection	8 (13.8)	7 (12.1)	0	< 0.001

a Mean ± SD.

**Table 2:** Mean ± SD, prevalence, and corresponding age-adjusted OR for hearing loss ≥25 dB in study groups by model.

		Model 1		Model 2^a^	
Exposure status	No.	Mean ± SD (%)^b^	OR (95% CI)^c^	Mean ± SD (%)^b^	OR (95% CI)^c^
Administrative	60	14.6 ± 6.01 (5.0)*	1.0	14.6 ± 6.4 (8.3)*	1.0
Noise only	58	26.2 ± 11.1 (44.8)*	15.4 (4.3 –54.9)	23.9 ± 11.8 (32.8)*	5.4 (1.8–15.6)
Toluene/noise^d^	58	29.8 ± 6.8 (86.2)*	119 (29.8–471)	27.7 ± 7.9 (67.2)*	22.6 (7.8–65.6)
33.0/83.2	13	30.9 ± 7.8 (84.6)	104 (15.6 –699)	28.1 ± 8.3 (76.9)	36.7 (7.5–178)
107.6/84.1	22	29.9 ± 7.9 (86.4)	120 (22.3–646)	28.1 ± 9.5 (72.7)	29.3 (7.9–109)
164.6/84.1	23	29.1 ± 5.1 (87.0)	127 (23.6 –678)	27.1 ± 6.0 (56.5)	14.3 (4.2–49.0)

a Pure tone of 0.5 kHz was excluded.

b Prevalence of hearing loss ≥25 dB.

c OR of hearing loss ≥25 dB.

d Toluene levels given in ppm, and noise levels given in dB(A).

* *p* < 0.001 for comparison between any two groups.

**Table 3:** Multivariate logistic regression analyses showing ORs (95% CIs) of hearing loss of ≥25 dB for toluene plus noise and noise-only groups.

		Model 1		Model 2^a^	
Variable	Sample size	*n*^b^	OR (95% CI)	*n*^b^	OR (95% CI)
Administrative	60	3	1.0	5	1.0
Noise-only	58	26	12.8 (3.4–47.6)	19	5.0 (1.7–15.1)
Toluene by CEI (year-ppm)	58	50	140 (32.1–608)	39	29.1 (9.3–91.4)
< 200	13	10	104 (15.2–713)	9	48.0 (9.2–252)
200–530	12	12	> 1,080 (313 to > 9,999)	9	55.6 (9.7–317)
531–2,000	15	13	102 (14.2–739)	11	30.4 (6.3–146)
≥2,001	18	15	92.8 (15.1–572)	10	14.3 (3.5–58.3)
Age (years)					
< 40	62	25	1.0	18	1.0
40–49	99	46	2.2 (0.8–6.1)	38	2.4 (0.9–6.2)
≥50	15	8	1.3 (0.2–7.3)	7	2.4 (0.5–11.1)
Smoking tobacco					
No	57	27	1.0	24	1.0
Yes	110	49	0.6 (0.2–1.6)	36	0.5 (0.2–1.2)
Quit	9	3	1.1 (0.2–6.5)	3	1.4 (0.3–7.3)
Drinking alcohol					
No	86	40	1.0	32	1.0
Yes	69	26	1.2 (0.4–3.1)	24	1.4 (0.6–3.2)
Quit	21	13	3.7 (0.9–14.8)	7	1.1 (0.3–3.4)
Use hearing protection					
Yes	15	12	1.0	8	1.0
No	161	67	0.3 (0.1–1.4)	55	0.7 (0.2–2.6)

a Pure tone of 0.5 kHz was excluded.

b Number of persons with hearing loss ≥25 dB.
